# Poromechanical controls on spontaneous imbibition in earth materials

**DOI:** 10.1038/s41598-021-82236-x

**Published:** 2021-02-08

**Authors:** Amir H. Haghi, Richard Chalaturnyk, Martin J. Blunt, Kevin Hodder, Sebastian Geiger

**Affiliations:** 1 Department of Civil and Environmental Engineering, University of Alberta, Edmonton, T6G 1H9 Canada; 2 Department of Earth Science and Engineering, Imperial College London, London, SW7 2AZ UK; 3 Institute of GeoEnergy Engineering, Heriot-Watt University, Edinburgh, EH14 4AS UK

**Keywords:** Hydrology, Solid Earth sciences, Energy science and technology, Engineering

## Abstract

Over the last century, the state of stress in the earth’s upper crust has undergone rapid changes because of human activities associated with fluid withdrawal and injection in subsurface formations. The stress dependency of multiphase flow mechanisms in earth materials is a substantial challenge to understand, quantify, and model for many applications in groundwater hydrology, applied geophysics, CO_2_ subsurface storage, and the wider geoenergy field (e.g., geothermal energy, hydrogen storage, hydrocarbon recovery). Here, we conduct core-scale experiments using N_2_/water phases to study primary drainage followed by spontaneous imbibition in a carbonate specimen under increasing isotropic effective stress and isothermal conditions. Using X-ray computed micro-tomography images of the unconfined specimen, we introduce a novel coupling approach to reconstruct pore-deformation and simulate multiphase flow inside the deformed pore-space followed by a semi-analytical calculation of spontaneous imbibition. We show that the irreducible water saturation increases while the normalized volume of spontaneously imbibed water into the specimen decreases (46–25%) in response to an increase in effective stress (0–30 MPa), leading to higher residual gas saturations. Furthermore, the imbibition rate decreases with effective stress, which is also predicted by a numerical model, due to a decrease in water relative permeability as the pore-space becomes more confined and tortuous. This fundamental study provides new insights into the physics of multiphase fluid transport, CO_2_ storage capacity, and recovery of subsurface resources incorporating the impact of poromechanics.

## Introduction

An accurate characterization of mechanical pore deformation, multiphase fluid transport, and their physical interactions (i.e., poromechanical interactions) in earth materials is essential for a diverse range of applications such as groundwater hydrology in the vadose zone^1,2^, geological CO_2_ sequestration^3,4^, transport of non-aqueous phase liquid contaminant in aquifers^5,6^, extraction of geothermal energy^7^, and enhanced oil recovery^8,9^. Recent experimental studies have revealed that the multiphase flow properties (e.g., relative permeability and capillary pressure) of rocks are dependent on effective stress-induced pore deformation^10–12^. Mechanistically, multiphase flow mechanisms in porous rocks are expected to be stress-dependent as well, an idea that challenges the simplifying assumption of a static pore structure addressed in numerous studies associated with multiphase flow in porous media^13,14^. Besides, models to forecast freshwater and hydrocarbon reserves and their recovery from subsurface formations need to include the complex interactions between poromechanics and multiphase flow^15,16^.

Every geological formation in the upper part of the earth’s crust is exposed to a state of compressive stress, which depends on depth, pore pressure, and active geological processes^17–19^. Geological processes (e.g., plate tectonics) shape the current state of in-situ stress and alter it slowly over geological timescales^20^. However, human-induced changes in effective stress, driven by excessive fluid production/injection operations in subsurface formations, occurs rapidly^20^. For instance, global overexploitation of groundwater from many giant aquifers, especially in northern China, India, Pakistan, Iran, and the United States (US), has caused vast land subsidence, fault reactivation, and induced seismicity^15,21–24^. In the US, 45 states with an area of more than 40,000 km^2^ have been affected by extensive land subsidence due to the compaction of aquifers and the collapse of cavities in carbonates^25^. Similarly, injection/production operations in hydrocarbon fields have led to an 80% loss of wetlands in Louisiana^20^. These are all large-scale consequences of significant human-driven changes in effective stress due to changes in pore pressure. Pore-spaces present within geological formations deform in response to the nontrivial changes in effective stress. The physical mechanisms controlling stress-dependent pore deformations are well-understood, both analytically (based on poroelasticity theory^26,27^), and experimentally^28^. These poromechanical interactions have been shown to have a major impact on single-phase (e.g., absolute permeability) and multiphase flow properties of porous media^10–12^. Hence, multiphase flow mechanisms including drainage (i.e., the wetting phase is displaced by the non-wetting phase) and imbibition (i.e., the non-wetting phase is displaced by the wetting phase) are also expected to be deformation-dependent in porous media. Primary drainage (PD) is defined as the first drainage process in a pore-space that is initially 100% saturated with the wetting phase^29^. Spontaneous imbibition (SI) refers to capillary-driven imbibition where the capillary number $Ca$ (i.e., a dimensionless ratio of viscous forces to capillary forces) is typically less than ${10}^{-5}$^29^.

Despite the wealth of studies on the environmental impact of effective stress-induced deformation in subsurface formations, the physical influence of pore deformation on PD and SI mechanisms in the unsaturated zones of aquifers, geological formations that are targets for carbon storage, and geothermal or hydrocarbon reservoirs remains unclear. Recent experiments using both pore-scale and core-scale analyses^10–12,30,31^ have featured the stress dependency of relative permeability and capillary pressure in different materials (e.g., carbonates and sandstones). However, the detrimental impact of effective stress-induced pore deformation on the reserves of depleted groundwater resources or energy recovery from geothermal and hydrocarbon reservoirs is yet to be fully explored.

To elucidate the fundamental physical interactions between effective stress-induced deformation and multiphase flow, we scan a water-wet carbonate core at zero confining stress (${\sigma }^{\prime}=0 MPa$) using X-ray computed micro-tomography (micro-CT). Then, we conduct a series of stress-dependent core-flooding experiments, PD and SI, in the same core. We combine conceptual proxy modeling (i.e., which we used to reconstruct a 3D stress-dependent pore-space model) and pore network modeling (i.e., which we used for pore network extraction and two-phase flow simulation) techniques to simulate stress-dependent PD and SI at the pore-scale and compare the results with the core-scale experiments. We find that the irreducible water saturation ${S}_{wir}$ (i.e., the normalized volume of the remaining water in the pore-space at the end of PD) increases with effective stress. We reveal that the deformation of pores and channels, in response to an increase in effective stress, hinders the advance of the invading gas phase during PD, while the gas injection pressure is fixed. We further observe that stress-dependent pore deformation induces a boost in the normalized volume (percent of pore volume) of the spontaneously imbibed water into the pore-space ${N}_{w}$, a decline in water relative permeability ${k}_{rw}$, and a rightward shift in gas relative permeability ${k}_{rg}$ during SI. These findings demonstrate the striking control of stress-dependent pore deformation on irreducible saturation during the PD-SI process in subsurface porous media and its drastic impact on our estimates of recoverable freshwater and energy resources and CO_2_ storage capacity in some formations.

### Core-scale experimental observations of stress-dependent fluid-fluid displacement

We used micro-CT to investigate the initial pore structure and distribution of the wetting and non-wetting phases (deionized water and N_2_, respectively) of an unconfined carbonate core (i.e., ${\sigma }^{\prime}=0 MPa$) at the end of PD and SI, independently. For the confined tests (i.e., ${\sigma }^{\prime}>0 MPa$), we mounted the specimen into a high-pressure, high-temperature triaxial cell to conduct a set of two-phase fluid flow experiments (the PD-SI process) under a wide range of isotropic effective stresses (1–30 MPa) and isothermal (40 ± 0.1 °C) conditions. The range of applied effective stress in the experiments was selected to cover the range during land subsidence due to aquifer depletion^25^ and compaction of hydrocarbon reservoirs in the US and the North Sea^20^ in response to human-induced effective stress changes ${\Delta \sigma }^{\prime}\le 30 MPa$. Changes in the applied effective stress on the specimen for each experiment was implemented by changing the confining pressure inside the cell while the pore pressure was kept constant. At each effective stress condition, we measured the pore strain and absolute permeability of the fully saturated specimen initially with deionized water. Effective stress-induced pore deformation drives a certain volume of water out of the fully-saturated specimen, which provided us with the pore strain ${\varepsilon }_{p}=({{V}_{p}}_{i}-\Delta V)/{{V}_{p}}_{i}$; where ${{V}_{p}}_{i}$ is the initial (i.e., unconfined) pore volume and $\Delta V$ is the directly measured volume of extracted water at each effective stress^27^. We calculated the absolute permeability, $k$ (${\mathrm{m}}^{2}$), by flushing deionized water at five flow rates ${Q}_{w}$ (1, 2, 3, 4, and 5 ml/min) through the saturated core. We then inserted the recorded pressure drop across the core, $\Delta p$, at steady-state condition into Darcy’s law $k={Q}_{w}{\mu }_{w}L/A\Delta p$, where ${\mu }_{w}$, $L$, and $A$ are defined as the water viscosity, characteristic length, and cross-sectional area of the core, respectively^32^. The PD-SI process consists of flushing the water-saturated core with N_2_ as the non-wetting phase and subsequently reintroducing water as the wetting phase. We conducted the PD and SI experiments at a constant injection pressure differential ($\Delta p$) of 500 kPa and 5 kPa, respectively, and atmospheric pore pressure under variable effective stress conditions. At each effective stress, the cumulative volume of the imbibed water was recorded every 20 s. In this study, we designed the experiments such that fluid-fluid displacements are capillary-dominant (i.e., spontaneous) imbibition with capillary numbers in the order of $Ca=({\mu }_{w}{v}_{w})/\gamma \approx {10}^{-8}$, where $\gamma$ is the interfacial tension, ${v}_{w}$ is the interstitial water velocity ${v}_{w}={Q}_{w}/(\varphi A)$, and $\varphi$ is the porosity of the rock^33^. More details on the core-scale experiments are outlined in “Materials and Methods” section.

### Stress-dependent pore flow modeling, σPFM

**Figure 1:**
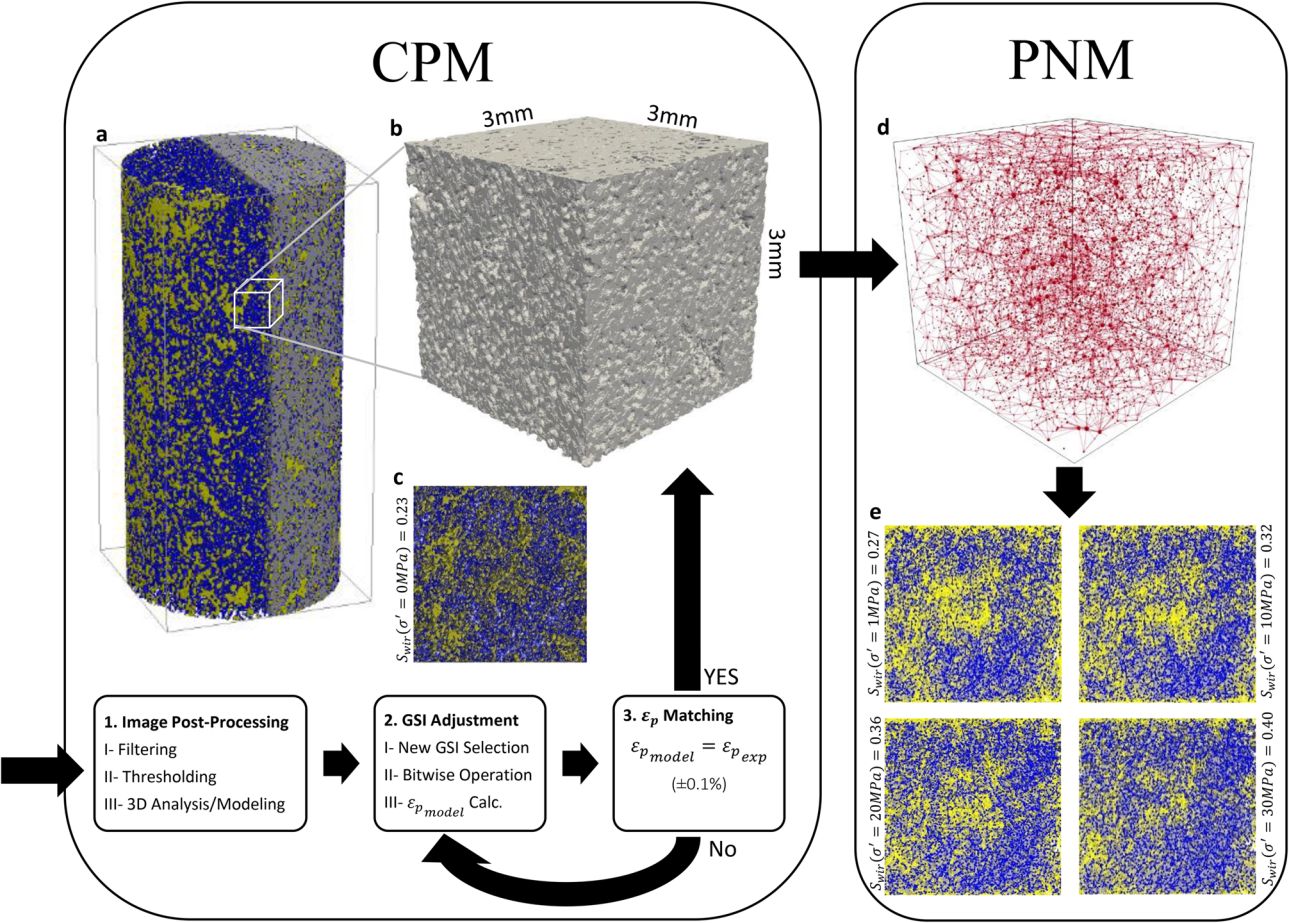
Workflow for stress-dependent pore flow modeling, σPFM. This figure shows (**a**) the 3D model of phase distribution in the unconfined core, (**b**) the cubic proxy model, (**c**) a 2D view of the phase distribution in the unconfined cube (blue is water and yellow is gas), (**d**) the stress-dependent extracted pore network model (PNM), and (**e**) 2D view of the simulated phase distribution in the cube (blue is water and yellow is gas) at ${S}_{wir}$ under increasing effective stress conditions. The conceptual proxy modeling (CPM) workflow consists of image post-processing, adjusting the grayscale index (GSI), and matching the proxy model’s pore strain (${{\varepsilon }_{p}}_{model}$) with the experimental pore strain (${{\varepsilon }_{p}}_{exp}$) at each effective stress condition.

We introduce the σPFM approach, which generates simultaneous visualizations of both stress-dependent pore structures and two-phase flow mechanisms in porous media, by coupling conceptual proxy modeling (CPM) and pore network modeling (PNM) techniques to quantify poromechanical impacts on fluid-fluid displacement in porous media (Fig. 1). The CPM technique is an efficient approach to compact the unconfined 3D pore-space model, which was extracted from the micro-CT images, and to reconstruct a proxy structure compatible with the pore-space at each effective confining stress condition (Fig. 1a–c^11^). Figure 1a shows the pore structure and qualitative distribution of the gas phase (in yellow) and the water phase (in blue) inside the unconfined core at the irreducible water saturation from segmented micro-CT images. To reduce the computational burden, we cropped a cubic sub-volume of the pore-space (Fig. 1b) with a size of 3 × 3 × 3 mm (350 × 350 × 350 voxels) out of the cylindrical core model (Fig. 1a) and used it in this study to reconstruct stress-dependent pore deformation. This cube reached the Representative Elementary Volume (REV) by performing REV analysis in Fig. S1 (see Supplementary Information) based on porosity (i.e., for a sub-volume, the REV has been reached once a plot of its porosity reached a plateau over different sample sizes). Meanwhile, the core and cube have the same porosity and absolute permeability. Figure 1c presents the phase distribution inside the unconfined cube at irreducible water saturation ${S}_{wir}({\sigma }^{\prime}=0 MPa)=0.23$. Next, we extracted a topologically representative network of the 3D deformed pore-space model (Fig. 1d), corresponding to each effective stress condition, and computed multiphase fluid transport via this network using an in-house code (Fig. 1e). A more detailed explanation of the σPFM approach is given in Supplementary Note 1.

### Analytical investigation of stress-dependent SI

According to the Lucas-Washburn law^34,35^, during SI, the location of the wetting-phase front scales with the square-root of time. Likewise, the normalized volume (percent of pore volume) of the spontaneously imbibed water, ${N}_{w}$, into the core follows the equation

1 $${N}_{w}=\frac{{\int }_{0}^{t}{q}_{wm}dt}{L\varphi }=\frac{2C}{L\varphi }\sqrt{t},$$

where ${q}_{wm}(m/s)$ and $t\left(\mathrm{s}\right)$ are defined as the maximum water invasion rate at the core inlet (${q}_{wm}=C/\sqrt{t}$) and time, respectively^36^. In Eq. (1), $C$ ($m/\sqrt{s}$) is a constant, which indicates the intrinsic potential of a porous media to imbibe the wetting phase spontaneously^37^. In this context, the principal governing equation for counter-current SI is defined as

2 $${F}_{w}\frac{{d}^{2}{F}_{w}}{d{S}_{w}^{2}}=-\frac{\varphi D\left({S}_{w}\right)}{2{C}^{2}},$$

3 $$D\left({S}_{w}\right)=-k\frac{{\lambda }_{w}{\lambda }_{g}}{{\lambda }_{w}+{\lambda }_{g}}\frac{d{P}_{c}}{d{S}_{w}}$$

where ${F}_{w}$, $D({S}_{w})$, $\lambda$, and ${P}_{c}$ are the capillary-driven fractional flow (${F}_{w}={q}_{w}/{q}_{wm}$), capillary dispersion coefficient with the unit of $({\mathrm{m}}^{2}/\mathrm{s})$, mobility ($\lambda ={k}_{r}/\mu$), and capillary pressure, respectively^36^. To solve Eq. (2), first, ${k}_{rw}$, ${k}_{rg}$, and ${P}_{c}$ as a function of ${S}_{w}$ and $k$ should be defined for the porous medium at each effective stress condition. Using Eq. (3), $D\left({S}_{w}\right)$ can be developed at each effective stress condition. Then, Eq. (2) can be solved iteratively for ${F}_{w}$ using an implicit integral or through a backward-differencing numerical approximation scheme to specify ${F}_{w}({S}_{w})$ and $C$^33^. More details on the derivation of the analytical solution are given in Supplementary Note 2. The stress-dependency of $C$ has been investigated analytically by Haghi et al.^12^. In the next section, we discuss the indispensable impact of stress-dependent pore deformation on $C$ at both microscopic and macroscopic scales.

## Results and discussion

### Core-scale observations

**Figure 2:**
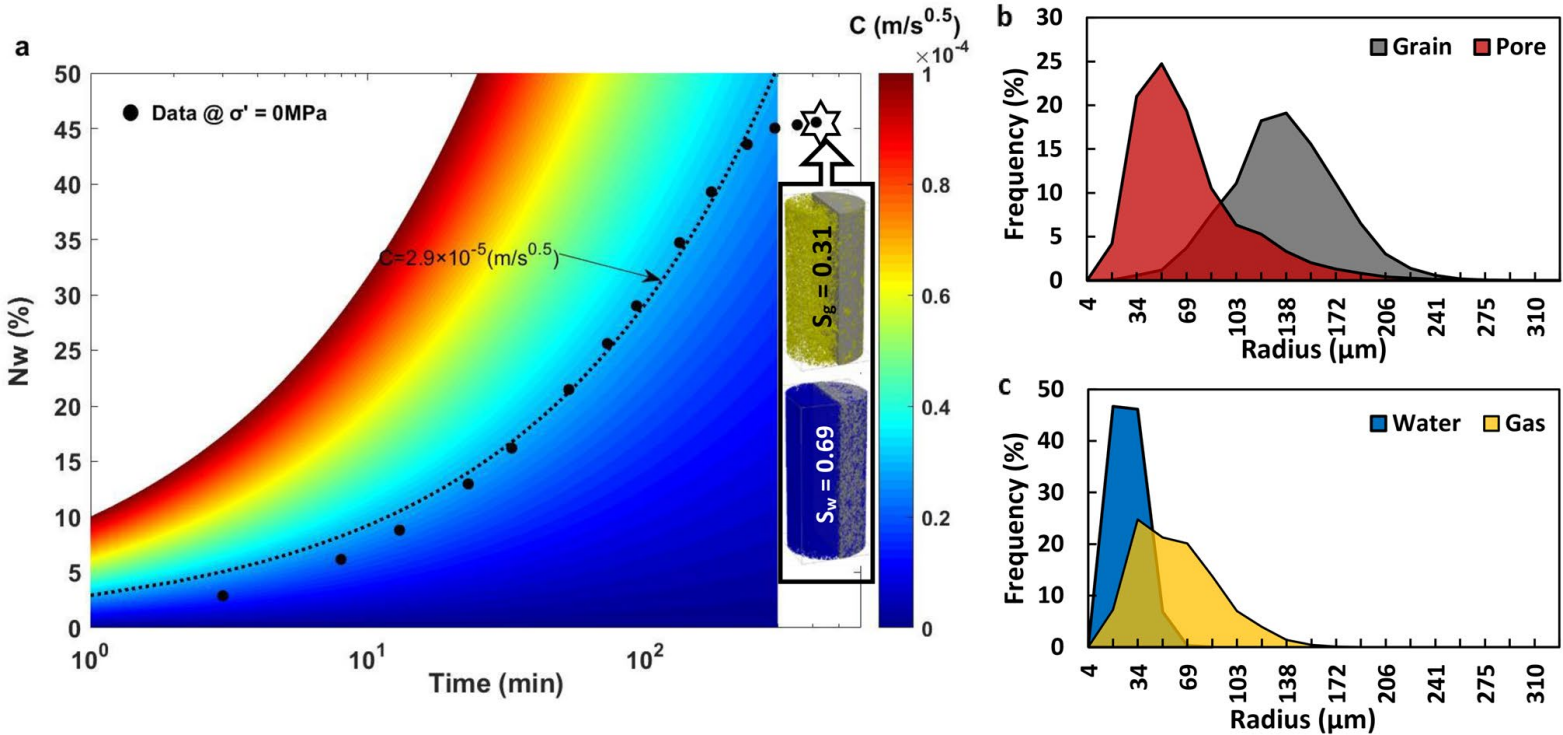
Quantitative insight into SI, phase distribution, and pore structure of the unconfined specimen. The plots show (**a**) the increment of ${N}_{w}$ over time with its corresponding $C$ value and the final saturation of water and gas phases, (**b**) the pore and grain size distribution, and (**c**) the wetting and non-wetting phase clusters size distribution after SI for the unconfined core.

Figure 2a presents the experimental data (black circles) of increments in ${N}_{w}$ over time on a semi-log plot with an ultimate ${N}_{w}=46\mathrm{\%}$ (i.e., where the data shows a plateau). Figure 2a shows that the curve with $C=2.9\times {10}^{-5} m/\sqrt{s}$ best matches our experimental data under a zero effective stress condition. Figure 2a further shows the gas and water saturation (0.31 and 0.69, respectively) and their qualitative distribution in the core at the end of SI. Quantitative descriptions of pore/grain structure and gas/water cluster size distributions are provided in Fig. 2b, c, respectively. Figure 2b indicates a pore and grain mean radius of 70 μm and 137 μm, respectively; both span up to 327 μm. Figure 2c shows that the gas phase is mainly trapped in the bigger pores (mean radius of 61 μm) while water occupies the small pores (mean radius of 28 μm) at the end of SI. Movie S1 (see Supplementary Information) shows the same SI of water, but at the scale of a single droplet at the surface of the unconfined carbonate specimen, which also reveals the strong affinity of the dry specimen to the water phase.

**Figure 3:**
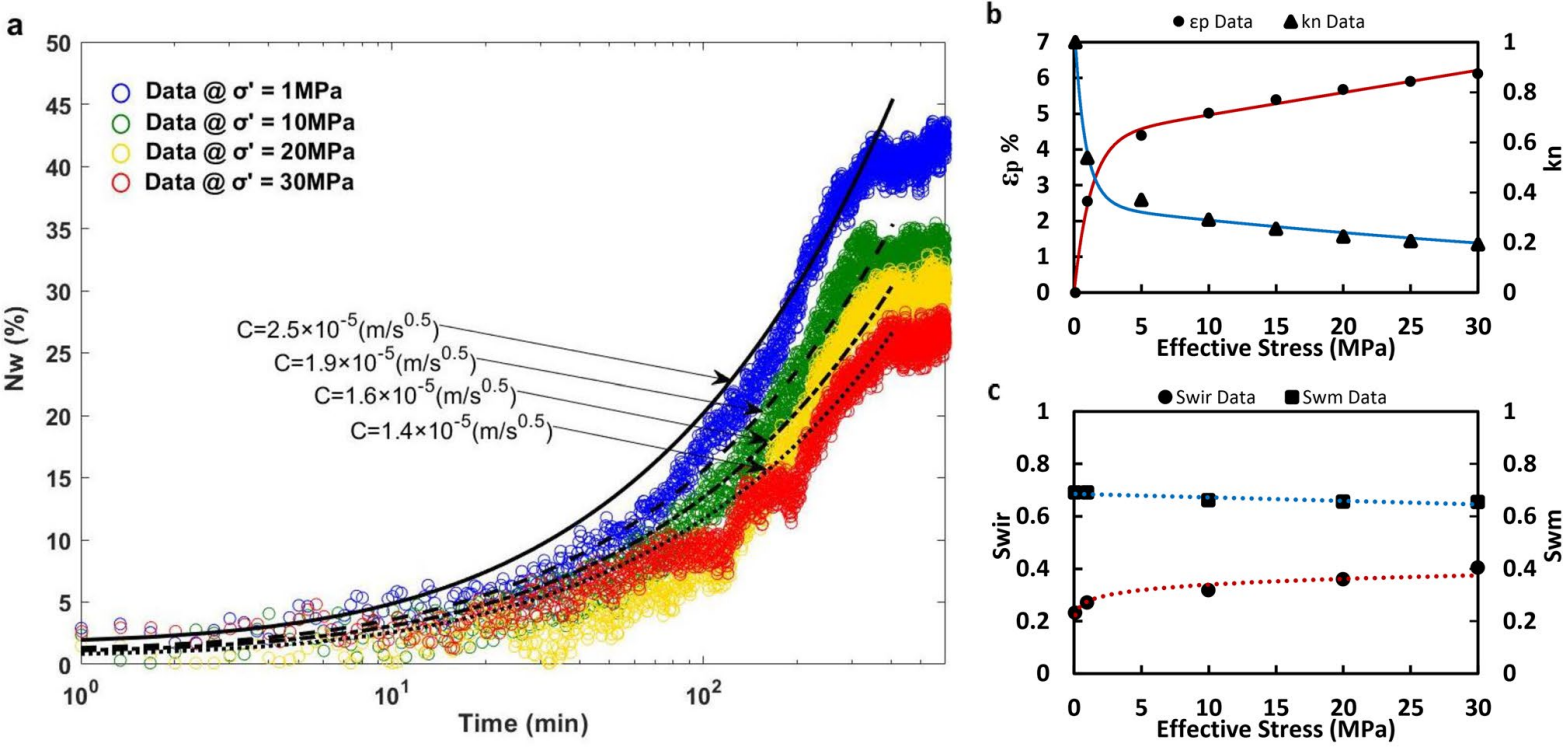
Characterization of stress-dependent SI, pore strain, absolute permeability, and end-point saturation using core-scale experiments. The figure provides plots of stress-dependent (**a**) ${N}_{w}$ over time with the corresponding $C$ values, (**b**) pore strain and normalized permeability, and (**c**) ${S}_{wir}$ and ${S}_{wm}$ at the core-scale.

The fitted curves in Fig. 3a were derived based on Eq. (1) and matched on the experimental ${N}_{w}$ data at four different effective stresses (1, 10, 20, and 30 MPa). Figure 3a shows a systematic decrease in the calculated $C$ ($2.5\times {10}^{-5}-1.4\times {10}^{-5}\mathrm{ m}/\sqrt{\mathrm{s}}$) and the measured ultimate ${N}_{w}$ (42–25%) by increasing the effective confining stress (1–30 MPa). These observations quantify the striking impact of poromechanics on fluid-fluid displacement, which is the main focus of this paper. Figure 3a further shows the trend of the decline in the calculated $C$ to be comparable with the intensity of changes in the pore strain; pore strain alteration attenuates drastically when the effective stress is increased from 0 to 10 MPa (Fig. 3b). Some experimental studies have revealed that ${N}_{w}\propto {\mathrm{t}}^{n}$ in a sparsely-connected pore-space ($n=0.5$ in Eq. (1)), where n depends on the pore connectivity (i.e., a poorly connected pore-space leads to $n<0.5$) and sample shape^38^. The value of $n$ might also increase by time for a single specimen until the wetting front exceeds the crossover length ${\chi }_{L}$ of percolation theory (i.e., accessible porosity to the core inlet decreases with distance until ${\chi }_{L}$ is exceeded)^38^. The variable $n$ value due to pore connectivity might also explain the slight initial deviation from the line of $n=0.5$ in Figs. 2a and 3a.

Figure 3b shows the alteration of pore strain (${\varepsilon }_{p}$) and normalized absolute permeability (${k}_{n}$) under an increasing state of effective confining stress. The blue curve in Fig. 3b was fitted on ${\varepsilon }_{p}$ data at each effective stress using the following non-linear equation (assuming pore strain is equal to volumetric strain^10^)

4 $${\varepsilon }_{p}=\frac{(1-{\gamma }_{S})}{{K}_{H}}\sigma {^{\prime}}-{\gamma }_{S}{e}^{-\sigma {^{\prime}}/{K}_{S}}+{\gamma }_{S}$$

where ${K}_{H}$ is the bulk moduli of hard parts (e.g., solid grain), ${K}_{S}$ is the bulk moduli of soft parts (e.g., pores), and ${\gamma }_{S}$ is defined as the fraction of (volume soft part)/(bulk volume) at the unstressed condition. Eq. (4) is derived based on natural-strain-based Hook’s law for the soft parts (i.e., an exponential function) and engineering-strained-based Hook’s law for the hard parts (i.e., a linear function)^10,39^. For the case of the current carbonate core, we calculated ${K}_{H}$, ${K}_{S}$, and ${\gamma }_{S}$ equal to 1.53 GPa, 1.25 MPa, and 0.043, respectively, using the least square regression method on eight ${\varepsilon }_{p}$ data-points (Fig. 3b) with ${R}^{2}=0.9941$.

We fitted ${k}_{n}$ data at each effective stress using the Carman and Kozeny correlation^40^, red curve, in Fig. 3b.

5 $${k}_{n}={\frac{k}{{k}_{i}}=\left(\frac{\varphi }{{\varphi }_{i}}\right)}^{3}{\left(\frac{1-{\varphi }_{i}}{1-\varphi }\right)}^{2}\left(\frac{1}{{\tau }_{n}}\right)$$

The index $i$ indicates the property at the unstressed condition. In Eq. (5), ${\tau }_{n}$ represents the non-linear normalized tortuosity of the flow path inside the porous medium $\stackrel{-}{\tau }=a({\left(1-{\varepsilon }_{P}\right)}^{-m}-1)+1$, where $a$ and $m$ are material constants^11^. Using the least square regression method, we calculated $a=0.68$ and $m=34.7$ for eight ${k}_{n}$ data-points (Fig. 3b) with ${R}^{2}=0.9966$. The calculated positive values for $a$ and $m$ can be translated into an increasing trend of ${\tau }_{n}$ in response to an increase in effective stress. Figure 3c outlines the sensitivity of the irreducible water saturation ${S}_{wir}$ and maximum water saturation ${S}_{wm}$ (i.e., maximum ${S}_{w}$ achieved at the end of SI) with effective confining stress. Figure 3c also represents a substantial increase in ${S}_{wir}$ (0.23–0.4) with an increase in effective stress (0–30 MPa), while the decrease in ${S}_{wm}$ is less significant (0.69–0.65).

In Fig. 3c, we find a significant increase in ${S}_{wir}$ with an increase in effective stress while injecting gas at a constant pressure differential ($\Delta p=500 kPa$) at all stress conditions. Our results from earlier stress-dependent core-flooding experiments showed a decrease in ${S}_{wir}$ with an increase in effective stress while injecting gas at constant flow rate (8 ml/min) at all stress conditions^10,11^. Hue and Benson^31^ argued that stress-dependent ${S}_{wir}$ in fractures is influenced strictly by the capillary number. Our findings support the same hypothesis in porous media using core-scale experimental results. Mechanistically, the compaction of the pore throats, due to an excess effective confining stress, obstructs the flow path of the invading gas phase during PD by increasing the flow channel’s capillary pressure, ${P}_{c}$, while the driving energy coming from the injection pressure is fixed. Based on the Young–Laplace equation^29^, ${P}_{c}=2\gamma \mathrm{cos}(\alpha )/r$, reducing the pore throat’s radius, $r$, leads to an increase in ${P}_{c}$ as long as interfacial tension and the contact angle, $\alpha$, are fixed. An increase in the channel’s capillary pressure in response to an increase in effective stress turns into a decline in capillary number, which significantly attenuates the capillary desaturation process of the water phase in the porous medium. This pore-scale logic explains why there is a continuum-scale increase in ${S}_{wir}$ by increasing the effective confining stress (Fig. 3c). Additionally, an increase in the channel’s capillary pressure due to stress-dependent compaction increases the likelihood of capillary instability and snap-off, which leads to an increase in the volume of the trapped gas in the porous rock (i.e., residual gas saturation, ${S}_{gr}$). This physical rationale elucidates the observed macroscopic decrease in ${S}_{wm}$ with an increase in effective stress in Fig. 3c. However, Fig. 3c shows that the stress dependency of the measured ${S}_{wm}$ (i.e., ${S}_{wm}=1-{S}_{gr}$) is not significant. This result can be explained by noticing the constant pressure differential ($\Delta p=5 kPa$) during SI was inadequate for significant mobilization of the trapped gas phase in the media, which implies that an increase in capillary number during the fluid-fluid displacement intensifies the poromechanical impacts on ${S}_{wir}$ and ${S}_{wm}$. For more clarifications on the two-phase experimental results, stress-dependent $C$ and effective saturation (${S}_{we}={S}_{wm}-{S}_{wir}$) are plotted in Fig. S2 (see Supplementary Information), which highlights a significant decline in both parameters in response to an increase in effective stress from 0 to 30 MPa.

### Pore-scale flow modeling

Using the σPFM approach, Fig. 1e reveals a systematic increase in the calculated ${S}_{wir}$ in response to an increase in effective stress, which is compatible with the experimentally measured values in Fig. 3c. Figure 1e further delivers qualitative insights into the distribution of the gas phase inside the cube, which is comparable with the observed gas distribution inside the cube at zero effective stress in Fig. 1c. In Fig. 1e, a manifestation of poromechanical controls on multiphase fluid flow at the pore-scale is the transition of the flow channels from a gas flow conduit to a gas capillary barrier by increasing the effective stress, which is evident through the dissipation of the region with a high gas concentration in the central part of the cube in response to stress-dependent pore deformation. Movies S2 and S3 (see Supplementary Information), which were developed from σPFM, present stress-dependent PD and SI in the cubic pore network model, respectively.

**Figure 4:**
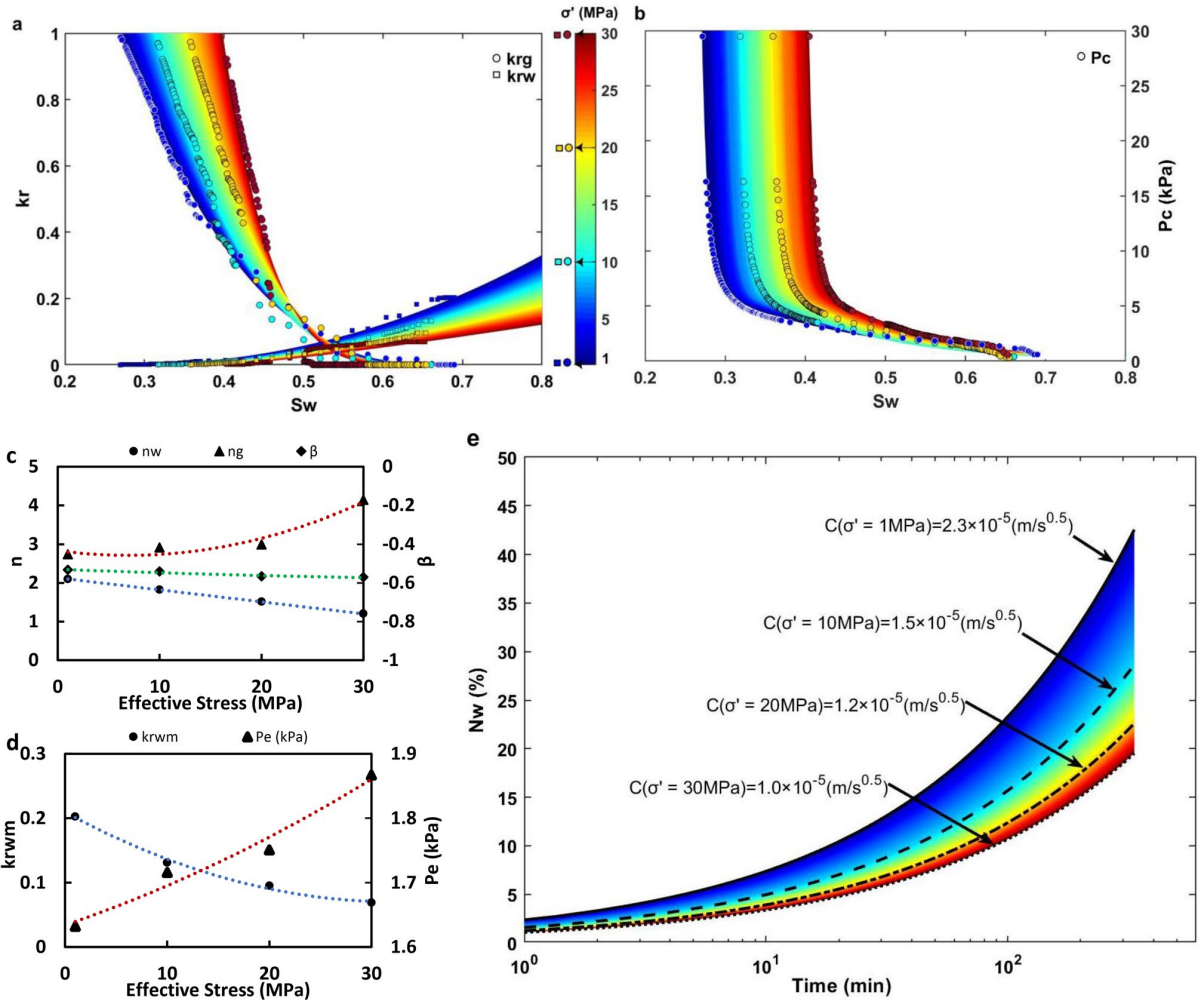
Pore-scale modeling results representing stress-dependent SI and multiphase flow properties of the cubic specimen using σPFM technique. This figure provides insights into stress-dependent (**a**) relative permeability ${k}_{r}$, (**b**) capillary pressure ${P}_{c}$, (**c**) fitting constants $n$ and $\beta$ in Eqs. (6–8), (**d**) curve fitting endpoints ${k}_{rwm}$ and ${P}_{e}$ in Eqs. (6–8), and (**e**) ${N}_{w}$ over time with the corresponding $C$ values for the cube.

Using σPFM, we provided a quantitative description of stress-dependent fluid-fluid displacement by deriving the multiphase flow properties of the network model, namely (1) SI relative permeability (Fig. 4a) and (2) SI capillary pressure (Fig. 4b), under increasing effective stress conditions. Figure 4a shows a rightward shift in ${k}_{rg}$, which is pertinent to our experimental observations associated with the increase of ${S}_{wir}$, in response to an increase in effective confining stress. Figure 4a also shows a decreasing trend in ${k}_{rw}$ with an increase in effective stress. This trend manifests the significant impact of pore-space deformation on flow-path tortuosity and flow conductivity during SI. We also added a plot of relative permeability versus $({S}_{w}-{S}_{wir})$ for comparison in Fig. S3 (see Supplementary Information) in which all relative permeability curves started from zero on the horizontal axis^41^. Moreover, true effective mobility function $TEM=k{k}_{r}/\varphi \mu$ versus $({S}_{w}-{S}_{wir})$ is also plotted in Fig. S4 (see Supplementary Information) as an alternative dynamic rock-typing process to quantify the impact of stress-dependent relative permeability on SI^42^. Both approaches reveal a systematic decrease in ${k}_{r}$ and $TEM$ function for water and N_2_ phases in response to an increase in effective stress, which indicates a decreasing trend of rock’s quality for water and N_2_ flow inside the specimen. Figure 4b reveals a significant increase in capillary pressure under increasing effective stress conditions, which is a hallmark of a pore network system under progressive compaction. In Fig. 4a,b, the 3D colored curves, which are extracted from the following power-law functions^10^, are fitted on the σPFM’s results (circles and squares). The color bar indicates variable effective stress conditions.

6 $${P}_{c}={P}_{e}{\left({S}_{w}^{*}\right) \, }^{\beta }$$

7 $${k}_{rw}={k}_{rwm}{\left({S}_{w}^{*}\right) \, }^{{n}_{w}}$$

8 $${k}_{rg}={k}_{rgm}{\left(1-{S}_{w}^{*}\right) \, }^{{n}_{g}}$$

In Eqs. (6–8), ${P}_{e}$ is the entry capillary pressure, ${k}_{rwm}$ is the maximum ${k}_{rw}$, ${k}_{rgm}$ is the maximum ${k}_{rg}$ (≈1 for imbibition), and ${S}_{w}^{*}=({S}_{w}-{S}_{wir})/({S}_{wm}-{S}_{wir})$. Furthermore, $\beta$, ${n}_{w}$, and ${n}_{g}$ are fitting constants (Fig. 4c). As illustrated in Fig. 4d, ${k}_{rwm}$ decreases and ${P}_{e}$ increases in response to stress-dependent pore compaction, which can be interpreted based on a decrease in the pore channel’s radius $r$ in the Carman-Kozeny model^40^, $k=\varphi {r}^{2}/8\tau$, and the Young–Laplace equation, respectively.

Figure 4e plots the calculated $C$ over time following the analytical framework given in the previous section and using the modeled relative permeability and capillary pressure with σPFM (Fig. 4a,b) under variable effective stress conditions. Figure 4e reveals a systematic increase in the calculated $C$ with an increase in effective stress, which is consistent with our experimental results (Fig. 3a) and a result of the decrease in water relative permeability with effective stress (Fig. 4a). Stress-dependent $C$ can be interpreted as the stress-dependent SI rate in geological formations. This observation demonstrates the success of the σPFM in the prediction of the poromechanical impact on fluid-fluid displacement in porous media. The slight inevitable difference between the predicted stress-dependent $C$ in Fig. 4e and the experimental values in Fig. 3a could be explained by scale effects (i.e., cube-scale versus core-scale) on the two-phase flow properties of porous media. In this study, the determination of whether our SI experiments in fact occurs by only co-current flow or a combination of co- and counter-current flow is not trivial. Experimental studies have shown that SI always has some degree of counter-current flow^43^. However, in this study, the presented analytical derivation for counter-current SI applies to the SI experiments because the displaced fluid (N_2_) is essentially inviscid and therefore all of the hydraulic resistance was in the imbibing water^44^.

**Figure 5:**
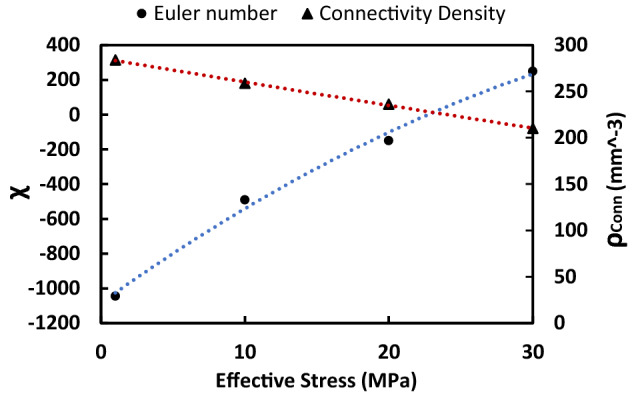
This figure shows stress-dependent changes in Euler number and connectivity density (mm^−3^) of the cube, which indicate the transition of the porous media from a well-connected ($\chi <0$) to a poorly-connected (χ $>0$) pore-space with an increase in effective stress from 0 to 30 MPa.

Figure 5 provides quantitative insights into changes in two topological invariants, namely (1) Euler Number ($\chi$), which is a measure of connectivity in the pore-space, and (2) connectivity density (${\rho }_{Conn}$), which indicates the number of redundant pore-throats per unit volume (${\mathrm{mm}}^{-3}$), with an increase in effective stress^45,46^. Details on these calculations are given in Supplementary Note 3. Figure 5 shows an increment in $\chi$ and a decline in ${\rho }_{Conn}$ in response to an increase in effective stress from 0 to 30 MPa, which are an indication of the transition from a well-connected ($\chi <0$) to a poorly-connected pore-space (χ $>0$).

### Applications in geoscience

Stress-dependent pore deformation due to fluid flow and change in pore pressure (i.e., poromechanics) is a ubiquitous phenomenon in nature^27^. Poromechanics has a prominent influence on fluid transport via living cells and tissues^47,48^, deformation of articular cartilage and cardiac muscles^49,50^, and plant movements^51^. Poromechanics is specifically a dominant mechanism for a wide range of applications in geoscience such as magma propagation in the earth’s mantle^47^. Human activities related to fluid withdrawal and injection operations in subsurface formations can significantly alter the state of in-situ stress in the earth’s crust, leading to reduced aquifer deliverability and surface subsidence, which has made the role of poromechanics in earth materials even more crucial. Induced seismicity and earthquakes triggered by stress changes are leading to deformation in groundwater systems and hydrocarbon reservoirs^52–54^. In multiphase flow systems, poromechanical interaction is important for many applications in groundwater hydrology, geophysics, and geoenergy, including water infiltration in the vadose zones, transport of non-aqueous phase liquid contaminant in aquifers, geological CO_2_ storage, energy extraction from geothermal reservoirs, and enhanced oil recovery.

In this study, we showed the striking impact of stress-dependent pore deformation on fluid-fluid displacement and the recovery of a carbonate specimen. N_2_ is an important gas in the atmosphere of the earth: dry air is composed of almost 80% nitrogen^55^. As gases are predominantly the non-wetting phase in most liquid-gas flows in natural porous media, the observed mechanisms for water-N_2_ from this study are extendable for a wider range of liquid-gas systems. In groundwater hydrology, water-air displacement is the governing fluid-transport mechanism in the dynamic unsaturated zones (i.e., vadose zones). Vadose zones have a great influence on the subsurface distribution of water^56^. Research has shown that 1.7 ± 0.4 billion people live in regions (dominated by the US, China, India, Iran, Saudi Arabia, Pakistan, and Mexico) where the global groundwater footprint ($GF$), (i.e., required aquifer area for a sustainable ecosystem and life) is greater than the actual area of hydrologically active aquifers ${A}_{a}$, with the current global $GF/{A}_{a}$ equal to $3.5\pm 0.7$^15^. Excessive groundwater abstraction causes aquifer depletion, an extension of the unsaturated zones in aquifers, and pore compaction due to an increase in effective stress. The evidence for this claim includes extensive land subsidence and sinkhole formation in these areas^25^. A significant reduction in the atmospheric emission of greenhouse gases is another global concern. Large-scale geological carbon storage is found to be essential to achieve this objective^57^. In this context, carbon capture and storage (CCS) is expected to reach a CO_2_ storage rate of 6000–7000 million tonnes per annum by 2050^58^. This highlights the importance of an accurate estimation of the CO_2_ storage capacity of the targeted subsurface formations.

We have shown that pore deformation due to an increase in effective stress from 0 to 30 MPa leads to a 75% increase (${S}_{wir}$=0.23 to 0.4) in the irrecoverable volume of water (irreducible water) in the carbonate sample after PD and a 46% decrease (${N}_{w}$=0.46 to 0.25) in the replenishable volume of pore-space with water. These findings highlight the critical role stress-dependent pore deformation plays in the calculation of the groundwater extent (i.e., available volume or thickness of groundwater) and ${A}_{a}$ in similar porous materials. Poromechanics controls not only the recoverable volume of groundwater during depletion, which is stored in the porous matrix of aquifers, but it also governs the ultimate volume and inflow rate of the groundwater into the porous matrix of unsaturated zones during the recharging phase, which may decelerate the aquifer replenishment process significantly. In a similar way, this dramatic change in ${S}_{wir}$, in response to an increase in effective stress from 0 to 30 MPa, can also be interpreted as a 22% decrease in the storable volume of gas inside the porous rock and the recovery of the liquid phase from the porous rock while the gas injection pressure is fixed. This dramatic change in the gas storage capacity and liquid recovery is critical for similar subsurface gas injection operations including CO_2_ geological storage and enhanced oil recovery (e.g., miscible/immiscible gas flooding), respectively. It is noteworthy that the considerable boost (46%) in ${N}_{w}$ during water imbibition with a decrease in effective stress conditions allows us to predict a surge in energy recovery from naturally fractured hydrocarbon reservoirs with a water-wet rock during water flooding. Although the carbonate sample used in this study does not represent all carbonates, this study highlights the significance of physical interaction between poromechanics and multiphase fluid flow in the generic porous media.

## Conclusions

We have explored the impact of stress-dependent pore deformation on multiphase flow mechanisms in a carbonate rock sample by conducting a set of isothermal PD and SI experiments under a wide range of effective confining stress conditions (0–30 MPa). We have shown that the irreducible water saturation ${S}_{wir}$ increases systematically when increasing effective stress (Fig. 3c), while injection pressure was fixed during PD. On the other hand, we have noticed a systematic decrease in the amount of water imbibed, ${N}_{w}$ and the maximum water saturation reached, ${S}_{wm}$ after SI in response to an increase in effective stress (Fig. 3). We have further shown the consistency between these core-scale stress-dependent observations with the non-linear changes in the pore strain and normalized permeability, where the rate of changes fades gradually by increasing effective stress conditions (Fig. 3b). Fitting Eq. (1) on the experimental ${N}_{w}$ data has proved the systematic dependency of $C$, which determines the SI rate, to effective stress conditions.

We have quantified the pore, grain, water cluster, and gas cluster size distribution of the unconfined specimen using micro-CT, which has provided us with insights into the initial structure of the pore-space and phase distribution (Fig. 2). We have further quantified stress-dependent changes in the topology of the pore-space using Euler number and connectivity density invariants, which demonstrated the transition of the porous media from a well-connected ($\chi <0$) to a poorly-connected (χ $>0$) pore-space with an increase in effective stress from 0 to 30 MPa. We have introduced the σPFM methodology by coupling CPM and PNM techniques to reconstruct the 3D stress-dependent pore-space model, extract the corresponding pore network model, and simulate stress-dependent PD and SI at the pore-scale (Fig. 1). Using σPFM, we have modeled stress-dependent relative permeability and capillary pressure for SI, which has predicted an increasing trend for ${S}_{wir}$ and ${P}_{c}$ and a decreasing trend for ${k}_{r}$ and TEM functions for both phases with an increase in effective stress condition (Fig. 4a,b). We have revealed that the analytical approach for SI based on σPFM’s results were successful in predicting the downward shift of ${N}_{w}$ under increasing effective stress conditions (Fig. 4e), which was compatible with the experimental data. This consistency between the core-scale experimental results and pore-scale predictions demonstrates that our method is reliable for stress-dependent multiphase flow modeling in deformable porous media.

We have discussed the poromechanical controls on multiphase flow mechanisms for a wide range of applications from biophysics to geoscience. We have shown that effective stress increase due to overexploiting groundwater threatens the sustainability of some groundwater resources and their dependent ecosystems. We have further shown the significant impact of pore deformation on the gas storage volume in CO_2_ sequestration and the recovery of hydrocarbon reservoirs. All these findings underscore the remarkable control of poromechanics on multiphase fluid flow in porous media and elaborate the physics behind the changes at the micro-scale and macro-scale, which pave the way for future relevant research in geoscience and engineering.

## Materials and methods

All the experiments were performed on a water-wet Indiana limestone sample (from Kocurek Industries INC., US) with a diameter of 3.73 cm, length of 10.1 cm, and initial density of 2260 kg/m^3^ (Fig. S5, Supplementary Information). Deionized water and ${N}_{2}$ were used as the wetting and non-wetting phases, respectively, with ${\mu }_{w}=652.7\mathrm{ \mu Pa s}$, ${\mu }_{g}=18.4\mathrm{ \mu Pa s}$, and $\gamma$=69.36 mN/m^59,60^. At the end of each stress-dependent PD-SI process, the core was flushed with CO_2_ to displace the trapped ${N}_{2}$ in the pore-space, vacuumed instantly over a liquid nitrogen cold trap to remove residual CO_2_ in the core, and re-flushed with high-pressure deionized water for sufficient time to reach a 100% saturation of the water. The schematic of the core-flooding equipment, which was designed for the stress-dependent PD and SI experiments, is shown in Fig. S5 (see Supplementary Information). The porosity and absolute permeability of the unconfined specimen were measured as 0.153 and $2.96\times {10}^{-14} {\mathrm{m}}^{2}$, respectively. The air-water contact angle (${\alpha }_{A-W}$) at the equilibrium condition was determined to be ≈ 60° using a Drop Shape Analyzer (DSA). This confirms the water-wet essence of the specimen being exposed to air as the second phase (Fig. S6, Supplementary Information). The measured ${\mathrm{\alpha }}_{\mathrm{A}-\mathrm{W}}$ was also used in the PNM code as the intrinsic contact angle. The unconfined core was initially scanned using micro-CT with a voxel size of 8.6 μm to capture the pore structure and phase distribution at ${S}_{wir}$ and ${S}_{wm}$. The carbonate core was contained inside a Viton rubber sleeve for all of the core-flooding experiments and stationary X-ray scanning processes. The full-diameter core was scanned in the Pharmaceutical Orthopedic Research Lab (PORL) at the University of Alberta using the micro-CT imaging suite. The voltage and spot size of the sealed tubal X-ray source within the imaging equipment was 100 kV and < 5 μm. The equipment provided a 360° rotational field-of-view. The post-processing and calculations were performed using the CT Analyser in Bruker micro-CT 3D Suite software. The workflow for the image post-processing is illustrated in Fig. S7 (see Supplementary Information). We performed skeletonization and sphere-fitting steps to derive the size distribution plots in Fig. 2 (Supplementary Note 1). In Figs. 2, 3 and 4, the least square regression method was used to fit curves on the experimental data and calculate constants.

## Supplementary Information

Supplementary Information.

Supplementary Movie S1.

Supplementary Movie S2.

Supplementary Movie S3.
