# Supplementary material for: Poromechanical controls on spontaneous imbibition in earth materials
Source: Sci Rep. 2021 Feb 8;11:3328. doi: 10.1038/s41598-021-82236-x (PMC7870954; doi:10.1038/s41598-021-82236-x)
Supplement: Supplementary file 1 — Supplementary Information. [file 41598_2021_82236_MOESM1_ESM.pdf]

Supplementary Information for

Poromechanical Controls on Spontaneous Imbibition in Earth Materials

Amir H. Haghi[1,*], Richard Chalaturnyk[1], Martin J. Blunt [2], Kevin Hodder[1], Sebastian Geiger[3]

[1]Department of Civil and Environmental Engineering, University of Alberta, Edmonton T6G 1H9, Canada.
[2]Department of Earth Science and Engineering, Imperial College, London SW7 2AZ, UK.
[3]Institute of GeoEnergy Engineering, Heriot-Watt University, Edinburgh EH14 4AS, UK.
*haghi@ualberta.ca

This PDF file includes:

- Supplementary Notes 1-3
- Supplementary Figures 1-7
- Supplementary Movies 1-3
- Supplementary References

Supplementary Note 1

Stress-Dependent Pore Flow Modeling, σPFM

Conceptual Proxy Modeling. The CPM technique starts with the X-ray micro-CT image post-processing and calculation for the unconfined specimen (i.e. under zero effective stress condition), which is elaborated in supplementary Figure 3 in detail. Matching the model's 3D porosity with the experimentally measured porosity at zero effective stress, we obtain the 3D model's pore volume at the initial condition, $V_{P_{Mi}}$. At each incremental effective stress condition, the CPM approach is pursued via these steps: 1) adjusting the micro-CT images' grayscale index, GSI, 2) assigning the model's pore-space as a 3D object through the bitwise operation, 3) calculating the 3D model's pore volume, V_{P_M}, and pore strain, $\varepsilon_{p_{model}}$, and 4) matching the 3D model's pore strain with its corresponding experimental value, $\varepsilon_{p_{exp}}$, through an iterative process. The step of GSI adjustment (no.1) acts similar to the erosion operation in morphological image processing (1). It is noteworthy that the erosion and changes in pore structure does not accumulated during the CPM iterations as the original binary image is reloaded at the beginning of each iteration. $\varepsilon_{p_{model}}$ is calculated using the equation

$$\varepsilon_{p_{model}} = \frac{(V_{P_{Mi}} - V_{P_M})}{V_{P_{Mi}}}. \quad \text{(S1)}$$

The iteration is continued until the following condition is met.

$$\left(\varepsilon_{p_{model}} - \varepsilon_{p_{exp}}\right) \leq \pm 0.1\% \quad \text{(S2)}$$

Then, the reconstructed stress-dependent proxy model is used for further 3D analysis and pore network modeling approaches. More explanations on the CPM method could be found in reference (2).

Pore Network Modeling. Extracting a topologically representative network of the pore-space is the first key step of the PNM workflow. This network provides quantitative insights into the volume, radius, area, length, and shape of the pores and throats and describes which pores are connected via which throats. Several approaches have been introduced so far for pore-space network extraction including the Grain-based, Erosion-dilation, and maximal-ball (or sphere-fitting) approaches (3-5). In this study, the maximal-ball algorithm is implemented in the code where the largest spheres define pores, should be entirely placed inside the pore-space and centered on each void voxel (5), and the strings of finer spheres connecting pores represent throats, which recognized from medial axes of the pore through skeletonization process (6). After extracting the network out of the pore-space images, we simulate fluid flow through the network employing a Stokes solver with the assumption of a capillary-dominated (quasi-static) flow (7). The applied governing equations for the flow and dispersion process are given below,

$$\nabla . \mathrm{v} = 0 \quad \text{(S3)}$$

$$\mu \nabla^2 \mathrm{v} = \nabla P \quad \text{(S4)}$$

$$\mathrm{v} = 0 \text{ on grain surfaces} \quad \text{(S5)}$$

where P, μ, and v are pressure, fluid viscosity, and velocity vector (8). An iterative algorithm, which is called Semi-Implicit Method for Pressure-Linked Equations (SIMPLE), is used to solve above equations (9). Molecular diffusion effect, which accounts for particles random jump after the advection, is also included in the flow simulation based on the following equation,

$$\lambda = \sqrt{6 D_m \Delta t} \quad \text{(S6)}$$

where λ, D_m, and Δt are defined as displacement, molecular diffusion coefficient, and time step size (10).

For any point in the pore-space, permeability of each phase is found using Darcy's law with the assumption of the independent flow of each phase. The simulation starts with the primary drainage process at 100% water saturation and followed by the imbibition process at irreducible wetting phase saturation. To determine receding and advancing contact angles, Morrow's class III model (11) is used. The readers are referred to the references (5) and (7) for more details on the pore network extraction and flow modeling techniques used in this study.

Supplementary Note 2

Analytical Solution for Capillary-Driven Imbibition

The conservation of mass law for incompressible water ($\rho_w = cte$) flow in a 1D two-phase flow system through a homogeneous porous medium leads to the following equation,

$$\varphi \frac{\partial S_w}{\partial t} + \frac{\partial q_w}{\partial x} = 0, \tag{S7}$$

which is known as the continuity equation in fluid dynamics (12). Applying Darcy's law for each phase linear flux, independently, and mixing both equations provide us with the following water Darcy velocity equation,

$$q_w = \frac{\lambda_w}{\lambda_t} q_t + k \frac{\lambda_w \lambda_{nw}}{\lambda_t} \frac{\partial P_c}{\partial x} - k \frac{\lambda_w \lambda_{nw}}{\lambda_t} \Delta\rho g_x \tag{S8}$$

where $\lambda = k_r/\mu$ is the mobility, $\lambda_t = \lambda_w + \lambda_{nw}$ is the total mobility, and g_x is the gravity component in the flow direction (11). Here, the index nw stands for the non-wetting phases. In equation (S8), q_w has three components including advection, gravity, and capillary. Rewriting equation (S8) for co-current spontaneous imbibition with the assumption of ignorable gravitational forces, we have (13)

$$q_w = f_w q_t - D(S_w) \frac{\partial S_w}{\partial x}, \tag{S9}$$

where the capillary dispersion coefficient is defined as

$$D(S_w) = -k \frac{\lambda_w \lambda_{nw}}{\lambda_t} \frac{dP_c}{dS_w}. \tag{S10}$$

Replacing q_w in equation (S7) with equation (S9) results in

$$\varphi \frac{\partial S_w}{\partial t} = -q_t \frac{df_w}{dS_w} \frac{\partial S_w}{\partial x} + \frac{\partial}{\partial x}\left(D(S_w) \frac{\partial S_w}{\partial x}\right) \tag{S11}$$

For the case of counter-current spontaneous imbibition, q_t in equation (S11) is equal to zero and hence we solve:

$$\varphi \frac{\partial S_w}{\partial t} = \frac{\partial}{\partial x}\left(D(S_w) \frac{\partial S_w}{\partial x}\right) \tag{S12}$$

Two parameters, namely 1) capillary fractional flow F_w and 2) similarity variable $\omega = x/\sqrt{t}$, have been introduced by McWhorter and Sunada (13) to find a mathematical solution for equation (S12), where ω and F_w were proved to be interrelated based on equation (S13).

$$\omega(S_w) = \frac{2C}{\varphi} \frac{\partial F_w}{\partial S_w} \tag{S13}$$

Substituting these two parameters in equation (S12) through some mathematical operation leads to the principal governing equation for the counter-current spontaneous imbibition (equation 2) with the conditions $F_w(S_{wir}) = 0$, $F_w(S_{wm}) = 1$, and $\omega(S_{wm}) = 0$ (12). Integrating equation (2) twice, we have

$$F_w = 1 - \left(\int_{S_w}^{S_{wm}} \frac{(\beta - S_w)D}{F_w} d\beta\right) . \left(\int_{S_{wir}}^{S_{wm}} \frac{(S_w - S_{wir})D}{F_w} dS_w\right)^{-1} \tag{S14}$$

and

$$C^2 = \frac{\varphi}{2} \int_{S_{wir}}^{S_{wm}} \frac{(S_w - S_{wir})D}{F_w} dS_w. \tag{S15}$$

Equation (S14) is an implicit integral, which needs an initial guess for F_w (e.g., $F_w = (S_w - S_{wir})/(S_{wm} - S_{wir})$, (14)). Calculation of F_w from the integral equation (S14) can be achieved by iteration until the difference between the successive approximates of F_w becomes negligible. Knowing F_w, the constant C can be then computed using equation (S15). In equation (2), F_w and C can also be approximated using a backward-differencing numerical scheme (15), which yields

$$\frac{d^2F_w}{dS_w^2} \approx \frac{F_w(S_w + 2\Delta S_w) - 2F_w(S_w + \Delta S_w) + F_w(S_w)}{\Delta {S_w}^2} \quad \text{(S16)}$$

Using both methods, we find the calculated value for the constant C compatible in this study (Fig. 4e).

Supplementary Note 3

Topology Analysis of Pore-Space

Computing topological invariants of the pore-space is an efficient method to describe the structural arrangement of pore and solid within porous media (16). In this study, stress-dependent topology is quantified via the Euler number χ (i.e., Euler-Poincare or Euler characteristics), which is specified as the sum of Betti (β) numbers:

$$\chi = \beta_0 - \beta_1 + \beta_2 \quad \text{(S16)}$$

where the basic topological properties β_0, β_1, and β_2 define as the number of isolated objects (i.e., pores) in the volume, the number of redundant connections within the objects (i.e., pore-throats in the pore-space), and the number of completely enclosed cavities (i.e., grains) within the objects (17-18). For the case of the topology of pores in natural porous media, β_2 is typically ignored since grains fully surrounded by pores are rare (18). Based on equation (S16), the Euler number quantifies the connectivity of the pore-space which yields negative values for well-connected pore-space ($\beta_0 < \beta_1$) and positive values for poorly-connected pore-space ($\beta_0 > \beta_1$).

Euler characteristic analysis also provides a measure of connectivity density ρ_{Conn} (mm^{-3}), which indicates the number of redundant pore-throats β_1 per unit volume.

$$\rho_{Conn} = \frac{\beta_1}{V_t} \quad \text{(S17)}$$

In equation (S17), V_t (mm^3) is the total volume of the porous media (VOI).

In this study, we implemented the 3D calculation of Euler number and connectivity density using the CT Analyser program within Bruker microCT 3D Suite Software. For more details, readers are referred to the software manual (https://www.bruker.com/products/microtomography.html).

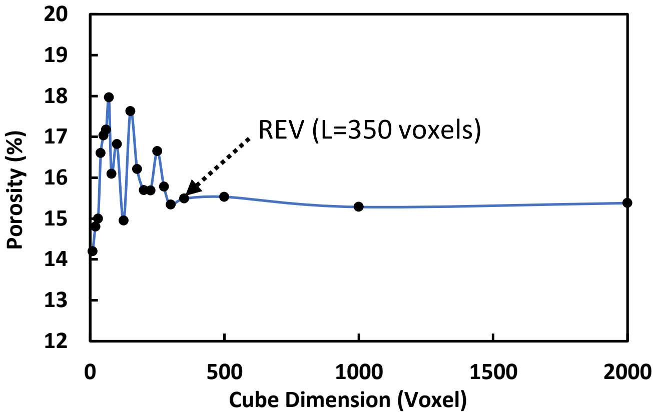

Figure S1. Determination of a cubic REV based on the porosity of the carbonate specimen. This plot demonstrates that a cube with a size of 350×350×350 voxels is reached REV as porosity is constant for cube dimension $L \geq 350$ voxels.

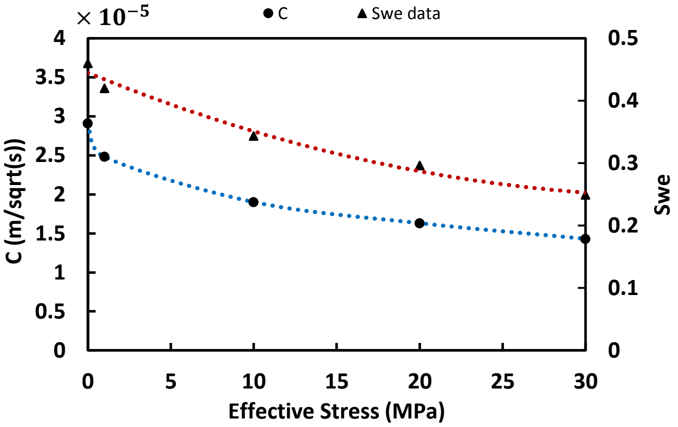

Figure S2. Characterization of stress-dependent C and S_{we} at the core-scale. This plot highlights the decreasing trend of C and S_{we} in response to an increase in the effective stress from 0 MPa to 30 MPa.

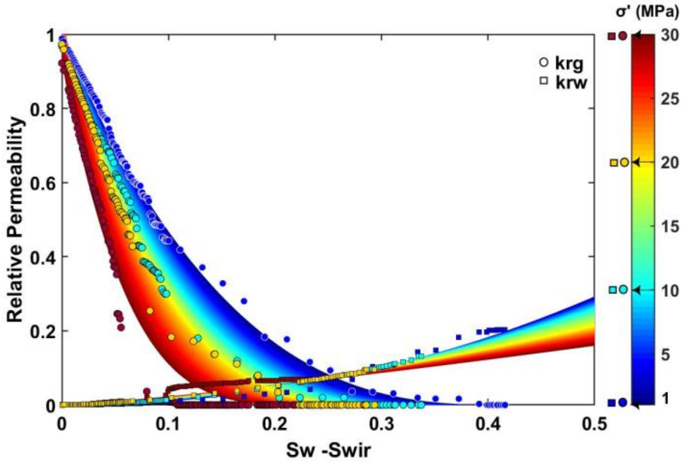

Figure S3. Pore-scale modeling results representing stress-dependent relative permeability k_r curves versus $(S_w - S_{wir})$. This plot reveals a decrease in k_{rg} and k_{rw} in response to an increase in effective stress from 1 MPa to 30 MPa by eliminating the impact of stress-dependent S_{wir} and shifting all curves leftward to zero.

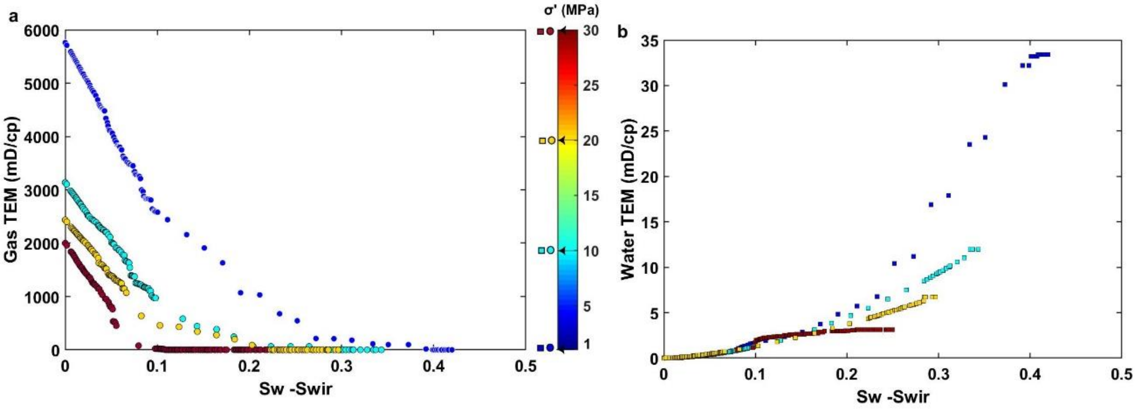

Figure S4. Pore-scale modeling results representing stress-dependent TEM-function versus $(S_w - S_{wir})$. This plot reveals a decrease in TEM-function for both a) gas and b) water phases in response to an increase in effective stress from 1 MPa to 30 MPa.

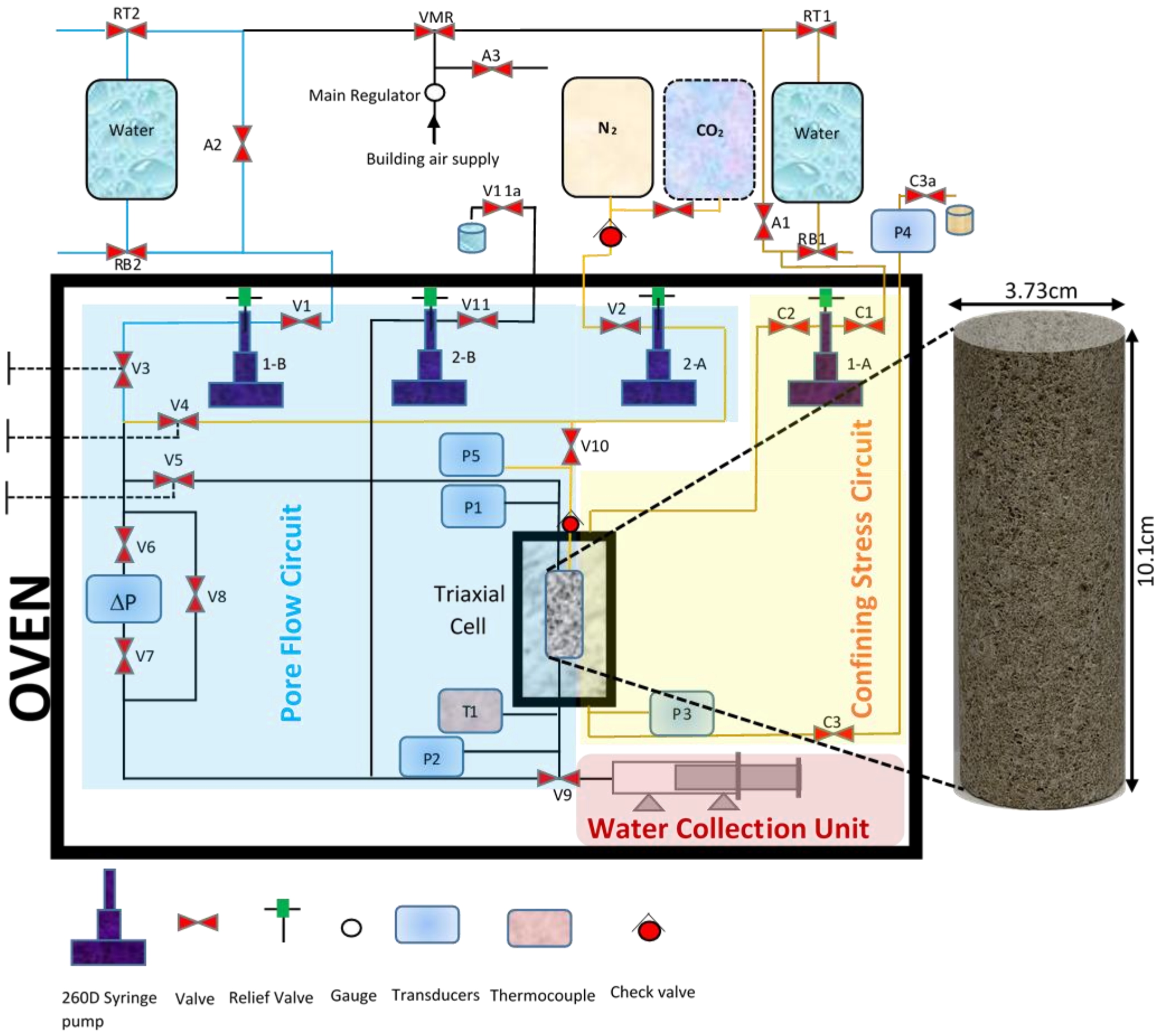

Figure S5. Schematic of core-flooding apparatus together with the photo of the carbonate core (modified from Haghi et al., (19)). The apparatus consists of three main units: 1) a multiphase pore flow circuit, 2) confining stress circuit, and 3) water collection unit. The pore flow circuit contains: 1) two syringe pumps (Teledyne ISCO 260D) for separate injection of gas and water phases into the specimen, 2) a syringe pump (Teledyne ISCO 260HP) to receive phases from the core outlet and maintain the pore pressure, and 3) couple of pressure transducers and thermocouples to record real-time pressure and temperature data, respectively. The confining stress circuit holds a high-pressure high-temperature triaxial cell, a syringe pump (Teledyne ISCO 260HP), and two pressure transducers. This circuit allows us to apply a wide range of effective confining stress on the specimen. The water collection unit is a graduated transparent syringe which is designed to collect the gas and water phases immediately from the core at the atmospheric pressure, separated them gravitationally, and evaluate accurately the weight and volume, $\boldsymbol{V_w}$, of the extracted water at the end of drainage process for $\boldsymbol{S_{wir}}$ calculation. Here, $\boldsymbol{S_{wir}} = (\boldsymbol{V_p} - \boldsymbol{V_w})/\boldsymbol{V_p}$, where $\boldsymbol{V_p}$ is the stress-dependent pore volume. All three units are entirely placed inside a spacious Dispatch LBB2 oven to conduct an isothermal (40^0C ± 0.1^0C) core-flooding experiment (19).

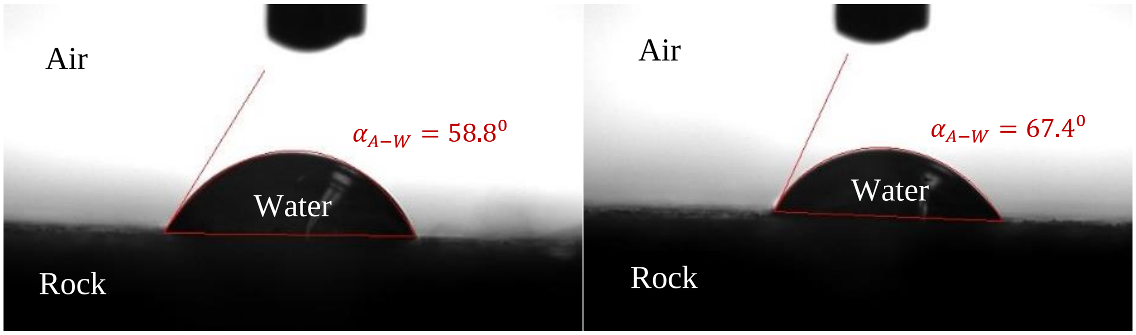

Figure S6. Measured air-water ($\boldsymbol{\alpha_{A-W}}$) contact angles using a Drop Shape Analyzer (DSA) at equilibrium condition. Based on the measured contact angle, the carbonate specimen is categorized as a water-wet rock in an air-water system ($\boldsymbol{\alpha_{A-W} < 90^0}$).

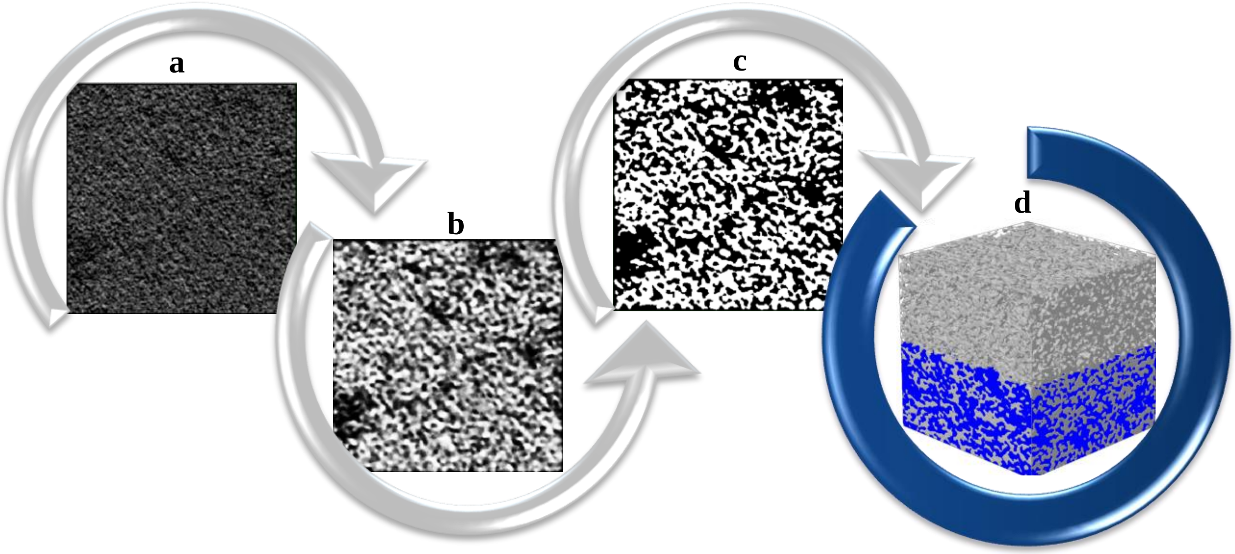

Figure S7. Workflow for X-ray micro-CT image post-processing and calculations for the unconfined specimen. The workflow consists of four main tasks: (a) uploading the raw images and selecting a square region of interest, (b) filtering, (c) thresholding and binarization, and (d) 3D analysis and modeling. For the filtering operation, we perform median and contrast enhancement steps to eliminate noises in the raw images. Filtered images are then binarized into black and white segments representing pores and grains, respectively, using Otsu's thresholding approach in 3D space (20). For the 3D analysis and modeling step, we create separate 3D models for grain and pore-space using bitwise operation. The water and gas phases are distinguished in the pore-space based on the sharp differences in their densities and corresponding grayscale indices. Experimentally measured porosity and phase saturations of the unconfined specimen are used to adjust the same parameters in the 3D model.

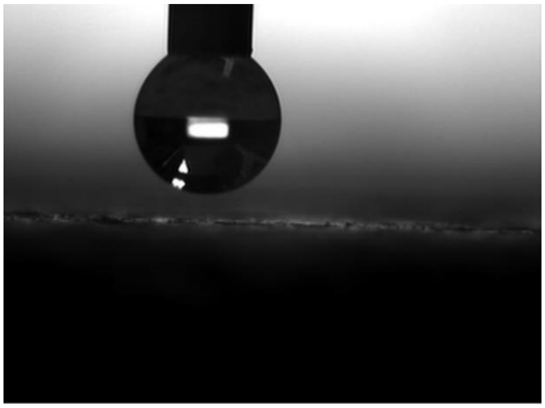

Movie S1 (separate file). Capillary-driven imbibition of water by the dry carbonate specimen. This short video depicts a high hydrophilic nature of the carbonate. The video is played at actual speed.

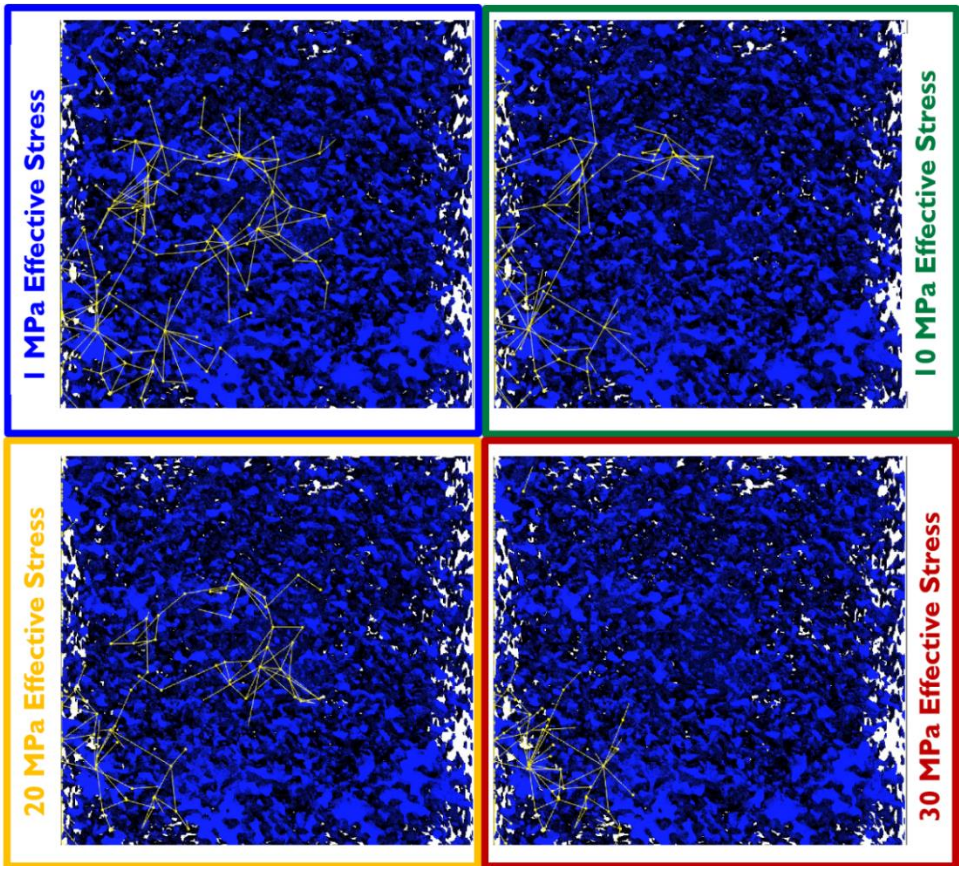

Movie S2 (separate file). Simulation of the primary drainage process through the pore space of the same carbonate specimen at four different effective stress conditions. Comparing these videos, we demonstrate visually the striking impact of the pore deformation on the flow path tortuosity, order of pores and throats that are invaded with the gas phase, and the saturation distribution across the cube. Here, the background images represent the stress-dependent water-saturated porous media and yellow ball and sticks indicate pore and throats, respectively, which are invaded with the gas phase.

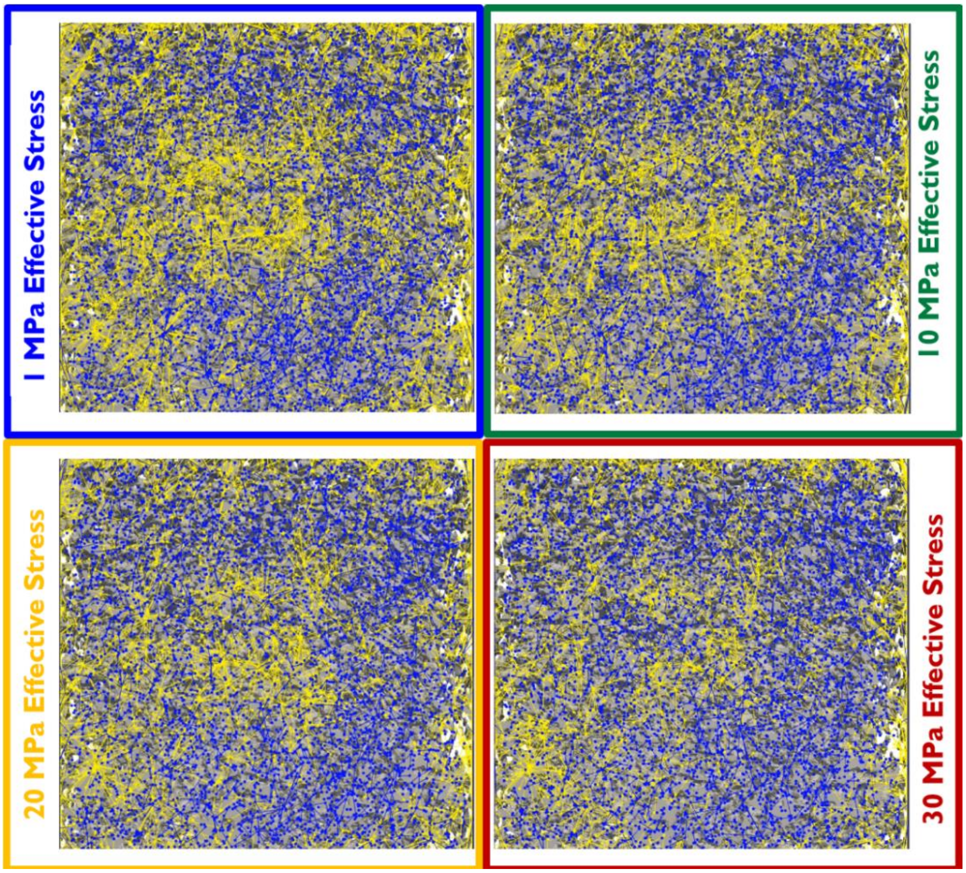

Movie S3 (separate file). Simulation of the imbibition process through the pore space of the same carbonate specimen at four different effective stress conditions. Comparing these videos, we manifest qualitatively the pore deformation induced-changes in the irreducible water saturation distribution and residual gas saturation distribution across the porous cube. Here, blue and yellow indicate water and gas phases, respectively. All four videos start with their corresponding pore-network at its stress-dependent irreducible water saturation condition and end at its maximum water saturation condition.

SI References

1. Efford, N. Digital image processing: a practical introduction using java (Addison-Wesley Longman Publishing Co., Inc, 2000).
2. Haghi, A. H., Chalaturnyk, R., & Talman, S. Stress-Dependent Pore Deformation Effects on Multiphase Flow Properties of Porous Media. *Scientific Reports* ***9*****(1)**, 1-10 (2019).
3. Øren, P. E., & Bakke, S. Process based reconstruction of sandstones and prediction of transport properties. *Transport in porous media* **46(2-3)**, 311-343 (2002).
4. Lindquist, W. B., Venkatarangan, A., Dunsmuir, J., & Wong, T. F. Pore and throat size distributions measured from synchrotron X-ray tomographic images of Fontainebleau sandstones. *Journal of Geophysical Research: Solid Earth* **105(B9)**, 21509-21527 (2000).
5. Dong, H., & Blunt, M. J. Pore-network extraction from micro-computerized-tomography images. *Physical review E* **80(3)**, 036307 (2009).
6. Silin, D., & Patzek, T. Pore space morphology analysis using maximal inscribed spheres. *Physica A: Statistical mechanics and its applications* **371(2)**, 336-360 (2006).
7. Valvatne, P. H., & Blunt, M. J. Predictive pore-scale modeling of two-phase flow in mixed wet media. *Water resources research* **40(7)** (2004).

8. Blunt, M. J., Bijeljic, B., Dong, H., Gharbi, O., Iglauer, S., Mostaghimi, P. & Pentland, C. Pore-scale imaging and modelling. *Advances in Water resources* **51**, 197-216 (2013).
9. Stüben K. A review of algebraic multigrid. J Comput. Appl. Math. **128**, 281–309 (2001).
10. Mostaghimi, P., Bijeljic, B., & Blunt, M. Simulation of flow and dispersion on pore-space images. *SPE Journal* **17(04)**, 1-131 (2012).
11. Morrow, N. R. The effects of surface roughness on contact: angle with special reference to petroleum recovery. *Journal of Canadian Petroleum Technology* **14(04)**, (1975).
12. Blunt, M. J. *Multiphase Flow in Permeable Media: A Pore-Scale Perspective*. (Cambridge University Press, Cambridge, 2017)
13. McWhorter, D. B., & Sunada, D. K. Exact integral solutions for two-phase flow. *Water Resources Research* **26(3),** 399-413 (1990).
14. Schmid, K. S., Geiger, S., & Sorbie, K. S. Semianalytical solutions for cocurrent and countercurrent imbibition and dispersion of solutes in immiscible two-phase flow. *Water Resources Research*, **47(2)** (2011).
15. Schmid, K. S., Alyafei, N., Geiger, S., & Blunt, M. J. Analytical solutions for spontaneous imbibition: fractional-flow theory and experimental analysis. *SPE Journal*, **21(06)**, 2-308 (2016).
16. Vogel, H. J. Topological characterization of porous media. In Morphology of condensed matter (pp. 75-92). (Springer, Berlin, Heidelberg, 2002).
17. H. Hadwiger, Vorlesung ¨uber Inhalt, Oberfl¨ache und Isoperimetrie, (Springer-Verlag, Berlin, 1957).
18. Herring, A. L., Robins, V., & Sheppard, A. P. Topological persistence for relating microstructure and capillary fluid trapping in sandstones. *Water Resources Research*, 55(1), 555-573 (2019).
19. Haghi, A. H., Talman, S., Chalaturnyk, R., Consecutive Experimental Determination of Stress-Dependent Fluid Flow Properties of Berea Sandstone and Implications for Two-Phase Flow Modeling. *Water Resources Research* ***56*****(1)**, (2020).
20. Otsu, N. A threshold selection method from gray-level histograms. *IEEE transactions on systems, man, and cybernetics* **9(1)**, 62–66 (1979).
